# Metabolic Regulation of Seasoned White Snakehead Fillets by a Lemon Essential Oil–Rutin–Chitosan Coating Under Controlled Freezing-Point Storage

**DOI:** 10.3390/foods15122091

**Published:** 2026-06-10

**Authors:** Jiaxin Han, Xuefei Luo, Lin Zhou, Qiaolan Zhu, Xinhui Wang, Jing Zhang, Bingliang Liu, Weijun Chen

**Affiliations:** Meat Processing Key Laboratory of Sichuan Province, College of Food and Biological Engineering, Food Security Publicity and Education Base of Sichuan Province, Chengdu University, Chengdu 610106, China; 15877423632@163.com (J.H.); 17721988832@163.com (X.L.); zhoulin94@cud.edu.cn (L.Z.); 212022095135178@cdu.edu.cn (Q.Z.); zhangjing2022@cdu.edu.cn (J.Z.); liubingliang@cdu.edu.cn (B.L.)

**Keywords:** white snakehead, coating, controlled freezing-point storage, untargeted metabolomics

## Abstract

This study evaluated how a lemon essential oil–rutin–chitosan coating (CS-LEO/NE-R), prepared from a 5:95 (v/v) lemon essential oil/rutin-containing nanoemulsion and a chitosan solution containing 1.5% chitosan, 1% acetic acid, and 5% glycerol, combined with controlled freezing-point storage preserves seasoned white snakehead fillets. Compared with controlled freezing-point storage alone, the combined treatment significantly inhibited oxidation, volatile nitrogen accumulation, texture softening, and microbial growth. On Day 10, the coating group recorded a total viable count of 4.98 log CFU/g, which was below the national limit (5 log CFU/g), whereas the control group went beyond this limit by Day 7. This extended the microbiological and physicochemical acceptability period by approximately 3 days under the present experimental conditions. Untargeted metabolomics revealed 2267 metabolites, and the differentially abundant ones mainly comprised amino acids, heterocyclic compounds, aldehydes, ketones, and esters. KEGG enrichment suggested that changes in linoleic acid metabolism, terpenoid related annotations, the actin cytoskeleton, and the phospholipase D signaling pathway were associated with delayed quality deterioration. This work provides a theoretical basis for the composite biopreservation of aquatic products.

## 1. Introduction

*Channa argus*, commonly known as northern snakehead, is an economically important freshwater fish species in China. In Sichuan, Chongqing, and other regions, it is also locally marketed as white snakehead because of its distinctive appearance and regional commercial naming practices. Owing to its tender texture, desirable flavor, and high contents of essential amino acids and unsaturated fatty acids, *Channa argus* has considerable nutritional and commercial value [1]. However, after death, fish muscle readily undergoes microbial contamination, proteolysis, and lipid oxidation, restricting its shelf life, distribution radius, and market development.

With increasing consumer demand and advances in aquatic product processing, the *Channa argus* industry is gradually shifting from traditional live-fish sales toward value-added processed products [2]. Seasoned white snakehead fillets have attracted growing market interest because they are convenient, ready-to-cook, and have relatively stable flavor characteristics. Nevertheless, processing does not eliminate quality deterioration during storage. These fillets remain prone to microbial growth, lipid oxidation, protein denaturation, and texture deterioration, all of which reduce product safety, sensory quality, and commercial value. Therefore, storage technologies that can better protect the quality of seasoned white snakehead fillets remain necessary [3].

Aquatic products are commonly preserved by chilled storage, freezing, modified-atmosphere packaging, chemical preservatives, or irradiation. Each approach has drawbacks: freezing can damage texture, chemical preservatives may reduce consumer acceptance, modified-atmosphere packaging increases cost, and irradiation may alter quality attributes [4]. Controlled freezing-point storage, which maintains products near their freezing point, can slow microbial growth and enzymatic reactions while reducing the formation of large ice crystals [5]. Although this approach is gentler than conventional freezing, its preservative effect may still be insufficient when used alone. Therefore, combining controlled freezing-point storage with active packaging or edible coatings has become a promising strategy for improving the storage stability of aquatic products [6]. Controlled freezing-point or ice-temperature storage has been applied to aquatic products such as processed grass carp products and channel catfish patties, where storage near the freezing point effectively delayed microbial growth, lipid oxidation, TVB-N accumulation, and protein degradation compared with conventional chilled storage [7].

Edible coatings based on chitosan have received extensive attention in food preservation because chitosan can form films, is biodegradable, and has antimicrobial activity. Han et al. previously designed a chitosan composite coating loaded with a lemon essential oil/rutin nanoemulsion, referred to as CS-LEO/NE-R. Through nanoemulsification, the formulation improved the stability and release behavior of lemon essential oil, which strengthened the antioxidant and antibacterial effects of the coating. When applied to pork, this system retarded lipid oxidation, microbial proliferation, and overall quality decline [8].

Based on the above work, the present research applied CS-LEO/NE-R coating in combination with controlled freezing-point storage to seasoned white snakehead fillets. Coated and uncoated samples were kept under the same near-freezing condition, and pH, texture, thiobarbituric acid reactive substances, total volatile basic nitrogen, and total viable counts were tracked during storage. Untargeted metabolomics was also conducted on selected samples to describe temporal metabolic changes and to relate them to deterioration. The objective was to determine whether CS-LEO/NE-R provides additional protection under controlled freezing-point storage and to offer mechanistic evidence for composite biopreservation of aquatic products.

## 2. Materials and Methods

### 2.1. Materials

Lemon essential oil (LEO), chitosan (CS), and rutin were purchased from Shanghai Yuanye Biological Co., Ltd., Shanghai, China. The white snakehead fillets selected for the experiments were bought from Shiling Vegetable Market in Chengdu, China. All other reagents were analytical grade unless otherwise stated.

### 2.2. Formulation of the CS-LEO/NE-R Coating

The coating solution was produced with reference to Han et al. (2025) [8]. In brief, lemon essential oil was added to a Tween-80 aqueous phase containing rutin to prepare the LEO/NE-R nanoemulsion at an oil-to-aqueous phase ratio of 5:95 (v/v), followed by homogenization and ultrasonication. The resulting nanoemulsion was blended with a chitosan solution containing 1.5% (w/v) CS, 1% (v/v) acetic acid, and 5% (w/v) glycerol, and the mixture was homogenized at 12,000 rpm for 4 min to obtain the final CS-LEO/NE-R coating.

### 2.3. Sample Preparation and Preservation

The fish fillets were cut to a thickness of 3.0–3.5 mm, and residual surface water was removed gently with sterile kitchen paper. Ultrasound-assisted marination was carried out in a solution containing 2% (w/w) composite phosphates and 4% (v/w) cooking wine, with a sample-to-marinade ratio of 1:3 (g/mL). A KQ3200DB digital ultrasonic cleaning bath (Kunshan Ultrasonic Instrument Co., Ltd., Kunshan, China) was operated at 40 kHz and 150 W. With a working volume of 6 L, the nominal power density was approximately 25 W/L. The fillets were completely submerged and treated for 30 min. During treatment, the marinade was monitored and kept at around 20 °C to reduce heat accumulation. The same ultrasound-assisted marination procedure was used for all samples to ensure comparable seasoning penetration before preservation.

After marination, samples were assigned to two treatments. Fillets in the coating group were dipped in the CS-LEO/NE-R solution for 30 s, while those in the control group were dipped in sterile distilled water for the same duration. The samples were then drained, wrapped with polyethylene film, placed on polyethylene trays, and then kept at −1 °C until analysis.

### 2.4. Freezing Point Detection

Following the methods of Fu et al. (2024) [9], the freezing point of seasoned white snakehead fillets was measured using an automatic temperature recorder (Testo Saveris 2, Testo AG, Lenzkirch, Germany). The probe was inserted into the muscle tissue, and the fillets were wrapped with polyethylene plastic wrap, sealed, and stored at −18 °C. Temperature readings were taken at 1 s intervals, with three replicates per sample.

### 2.5. pH Detection

The pH was determined according to Peng et al. (2024) [10]. Briefly, a mixture of 5.0 g of minced sample and 50 mL deionized water was prepared in a conical flask, stirred thoroughly, and allowed to stand for 30 min. After filtration, the supernatant was analyzed with a portable Testo 205 pH meter (Testo AG).

### 2.6. Texture Analysis (TPA)

TPA was performed as described previously Fu et al. (2024) [9], with slight modifications. Specimens measuring 5 cm × 5 cm × 0.5 cm were compressed under TPA mode on a TA-XT plusC texture analyzer (Stable Micro Systems, Godalming, UK) using a P/36R cylindrical probe (36 mm diameter).

### 2.7. Thiobarbituric Acid Reactive Substances (TBARS) Determination

The determination was performed following the methods of Chen et al. (2023) with slight modifications [11]. Briefly, 5 g of minced fillet was mixed with 50 mL of TCA mixture in a conical flask and shaken at 50 °C for 30 min using a THZ-82 oscillator (Changzhou Aohua Instrument Co., Ltd., Changzhou, China). After cooling, the mixture was filtered twice. Then, 5 mL of the filtrate was reacted with 5 mL of TBA solution at 90 °C for 40 min, and the absorbance of the reaction mixture was measured at 532 nm and 600 nm. Each group was tested in triplicate, and the mean value was calculated.

### 2.8. Total Volatile Basic Nitrogen (TVB-N) Determination

TVB-N was measured using GB 5009.228-2016 [12]. After adding 1 g/L magnesium oxide suspension, approximately 5.0 g of homogenized sample was subjected to extraction and steam-distillation. Volatile nitrogen compounds were trapped in 20 g/L boric acid absorption solution and then titrated with 0.01 mol/L hydrochloric acid. TVB-N was calculated from the hydrochloric acid volume consumed and expressed as mg/100 g.

### 2.9. Total Viable Count (TVC) Analysis

The TVC was evaluated using the plate count method described by Chen et al. (2023) [11]. The fillets were sliced into 5 g pieces and transferred into 90 mL of sterile distilled water. After thorough homogenization, serial dilutions of the resulting suspension were prepared for microbial enumeration on plate count agar (PCA). All operations were performed under aseptic conditions. Each sample group was tested in triplicate, and the results were expressed as log CFU/g.

### 2.10. Sensory Evaluation

Sensory assessment was adapted from Zhang et al. [13], with slight modifications. Five sensory attributes, including color and appearance, odor, surface condition, texture/elasticity, and overall acceptability, were evaluated during storage. A five-point descriptive scale was used for each attribute, in which 5 indicated excellent quality, 3 indicated the minimum acceptable quality, and 1 indicated unacceptable quality. The sensory panel consisted of 10 trained postgraduate students from Chengdu University who were familiar with aquatic product quality evaluation. Before the formal evaluation, all panelists were briefed on the evaluation procedure and scoring criteria.

To reduce subjective bias, each sample was assigned a randomly generated three-digit code and served to the panelists in a randomized sequence. Each panelist independently assessed the samples under the same conditions. Color and appearance, odor, and surface condition were evaluated by visual and olfactory observation, while texture/elasticity was assessed by gentle pressing. Because the samples were raw seasoned aquatic products, tasting was not performed for safety reasons. The final sensory score for each attribute was expressed as the mean score of all panelists.

The sensory test followed academic ethical requirements and was approved by the College of Food and Biological Engineering, Chengdu University (Approval No. CDUSS20260102). All panelists were informed of the evaluation procedure and provided consent before participation.

### 2.11. Nontargeted Metabolomics Analysis

Nontargeted metabolomics was performed with reference to a previously published protocol [14], with minor changes. After pulverization under liquid nitrogen, 20 ± 1 mg of sample powder was mixed with 400 μL of ice-cold 70% methanol containing internal standards. The mixture was vortexed (1500 rpm, 5 min), chilled on ice (15 min), and then centrifuged at 12,000 rpm for 10 min at 4 °C. The resulting supernatant was kept at −20 °C for 30 min and subjected to a second centrifugation under the same speed and temperature for 3 min. An aliquot of 200 μL of the final supernatant was transferred into an injection vial for UPLC-MS/MS analysis.

A Vanquish UHPLC system (Thermo Fisher Scientific, Waltham, MA, USA) equipped with a Waters ACQUITY Premier HSS T3 column (1.8 μm, 2.1 mm × 100 mm; Waters Corporation, Milford, MA, USA) was used for chromatographic separation. The mobile phases were water containing 0.1% formic acid and acetonitrile containing 0.1% formic acid. The column temperature was 40 °C, the flow rate was 0.4 mL/min, and the injection volume was 4 μL. MS detection was performed on a Q Exactive HF-X mass spectrometer (Thermo Fisher Scientific, Waltham, MA, USA) in both positive and negative electrospray ionization modes over an m/z range of 75–1000.

Quality assurance was maintained during the metabolomics run. Before data acquisition, the mass spectrometer was calibrated according to the manufacturer’s protocol to ensure mass accuracy and stable performance. QC samples were generated by pooling equal volumes of all sample extracts and injected regularly throughout the sequence. Internal standards in the extraction solvent were used to check extraction efficiency and instrumental response. Features showing more than 50% missing values or a coefficient of variation above 30% in QC samples were removed. QC-based support vector regression was then applied to correct signal drift and batch effects before statistical analysis.

Metabolites were identified by comparing accurate mass, retention time, and MS/MS spectra with an in-house library and public databases, including HMDB, METLIN, and KEGG. After log2 transformation and mean centering, principal component analysis and orthogonal partial least squares-discriminant analysis (OPLS-DA) were carried out in R software (version 4.1.2). Model reliability was checked by 200 permutation tests, and R^2^X, R^2^Y, Q^2^, and permutation *p* values were reported to evaluate potential overfitting. Differential metabolites were selected using VIP > 1 and *p* < 0.05, followed by KEGG annotation and enrichment analysis.

### 2.12. Statistical Analysis

All assays were conducted in triplicate, and results are shown as mean ± standard deviation. SPSS 27.0 was used for two-way ANOVA to test the effects of treatment, storage time, and their interaction on physicochemical, microbiological, textural, and sensory variables. Normality and variance homogeneity were checked before ANOVA. When sensory data did not satisfy parametric assumptions, non-parametric tests were used to compare groups at the same storage time. Statistical significance was set at *p* < 0.05.

## 3. Results and Discussion

### 3.1. Freezing Point Temperature of Seasoned White Snakehead Fillets

**Figure 1:**
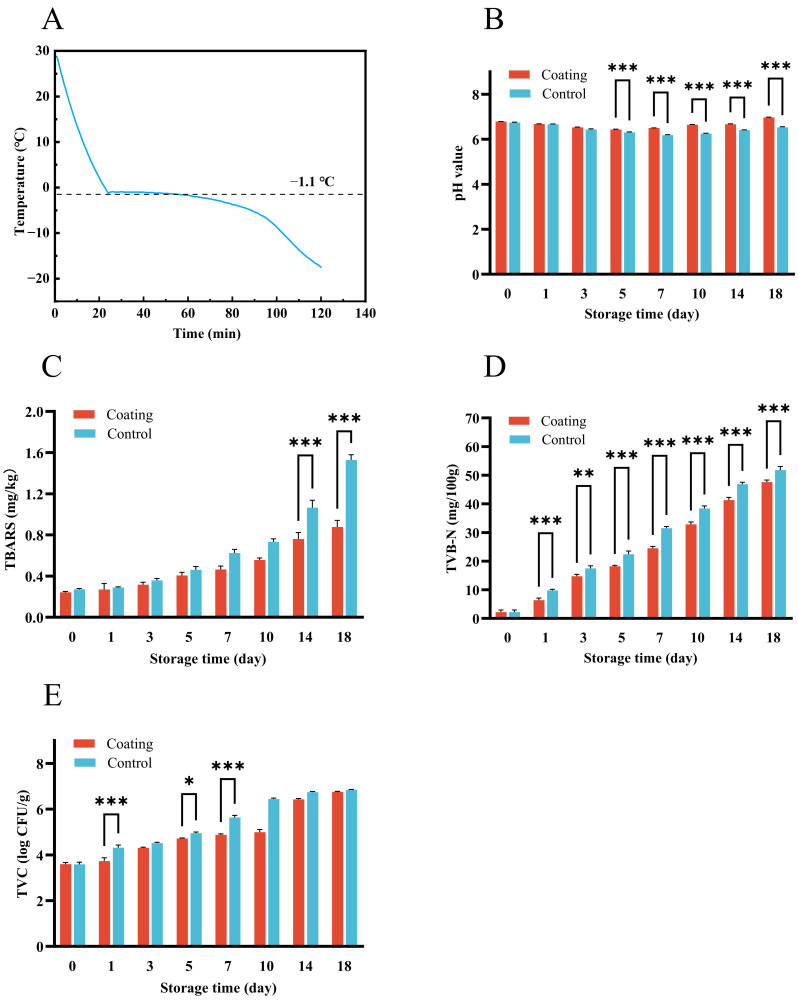
(**A**) Freezing temperature profile of seasoned white snakehead fillets. The blue solid line represents the temperature–time cooling curve, and the black dashed line indicates the determined freezing point of the samples (−1.1 °C). (**B**) pH, (**C**) TBARS, (**D**) TVB-N, and (**E**) TVC values of seasoned white snakehead fillets during storage. * indicates a significant difference at *p* < 0.05, ** indicates a significant difference at *p* ≤ 0.01, *** indicates a significant difference at *p* ≤ 0.001. Asterisks indicate significant differences between the control and coating groups at the same storage time based on post hoc comparisons after two-way ANOVA.

The freezing temperature curve of seasoned white snakehead fillets is shown in Figure 1A. During the initial freezing stage, the core temperature decreased from 29.0 °C to −0.1 °C over 22 min. Subsequently, from 22 to 31 min, the freezing curve plateaued, with the first inflection point observed at −1.1 °C. This phase corresponds to the maximum ice crystal generation zone, characterized by a marked reduction in the temperature change rate. Considering both the temperature control capability and the freezing point, −1 °C was ultimately selected as the controlled freezing-point storage temperature.

### 3.2. pH

The pH values of the seasoned white snakehead fillets in both groups initially decreased but then increased during storage (Figure 1B), consistent with the findings of Lin et al. (2025) [15]. The initial decrease was attributed primarily to lactic acid production from postmortem glycogenolysis and organic acids generated from ATP decomposition [16]. In the control group, the minimum pH value occurred on Day 5, followed by an increase to 6.51 by Day 7. For the coating group, the lowest pH was observed on Day 7 and then increased to 6.25 by Day 10. This increase was due mainly to alkaline amine compounds from protein decomposition by spoilage microorganisms [15]. Moreover, compared with the control group, the coating group exhibited a significantly slower rate of change in pH (*p* < 0.05), indicating that this treatment effectively inhibited microbial and enzymatic activity, thereby delaying the increase in pH.

### 3.3. TPA

Texture properties are critical for evaluating the quality and consumer acceptability of aquatic products [17]. As shown in Table 1, the hardness of seasoned white snakehead fillets significantly (*p* < 0.05) decreased as the storage time increased during controlled freezing-point storage, which was attributed mainly to protein degradation and hydrolysis by endogenous enzymes during storage. The more pronounced decrease in hardness observed in the control group was likely due to a greater extent of protein oxidation [18]. The initial springiness of seasoned white snakehead fillets was 0.62. After 18 days of storage, springiness decreased by 29% and 6.5% in the control and coating groups, respectively. Springiness primarily depends on the structural integrity of the muscle tissue, and prolonged storage allows for enzymatic and microbial activities to disrupt this structure, leading to reduced springiness [19].

Notably, both cohesiveness and resilience showed a transient increase on Day 1, followed by a progressive decline during subsequent storage. For example, in the control group, cohesiveness increased from 0.65 to 0.76, and resilience increased from 0.54 to 0.70 on Day 1. This initial increase may be attributed to short-term water redistribution, salt-induced swelling of myofibrillar proteins, and partial solubilization or rearrangement of the protein matrix in the seasoned fillets, which temporarily enhanced the compactness and recoverability of the muscle structure. Similar early-stage improvements in texture-related properties have been reported in lightly salted or coated fish fillets, where improved water-holding capacity and myofibrillar protein rearrangement contributed to delayed texture softening [20]. However, with prolonged storage, endogenous proteolysis, microbial activity, moisture migration, and protein oxidation progressively weakened the internal muscle network, leading to the subsequent decreases in cohesiveness and resilience [19,21].

Furthermore, chewiness significantly decreased (*p* < 0.05) during storage. Because chewiness is a comprehensive textural parameter related to hardness, springiness, and cohesiveness, its reduction directly indicates deterioration in the eating quality of the product [22]. Although cohesiveness and resilience temporarily increased on Day 1, the overall texture profile showed a downward trend over storage (*p* < 0.05). This trend was mainly associated with protein breakdown, lipid oxidation, moisture redistribution, and gradual disruption of muscle structure. Relative to the control, the coating partly alleviated these changes, indicating better maintenance of structural integrity in seasoned white snakehead fillets stored at the controlled freezing point. Texture measurements were extended to Day 18 to capture the full structural deterioration pattern, whereas practical acceptability was judged mainly by microbiological and chemical spoilage criteria.

### 3.4. TBARS

TBARS is commonly used to assess lipid oxidation in meat and fish products [23]. Figure 1C shows the TBARS changes in the two treatments. Values increased significantly in both groups throughout storage (*p* < 0.05), largely because lipid hydroperoxides and secondary oxidation products accumulated during unsaturated fatty acid oxidation [24]. The initial TBARS values were recorded as 0.27 mg/kg for the control group and 0.24 mg/kg for the coating group. By Day 18, these values increased to 1.53 mg/kg and 0.87 mg/kg, respectively. At this point, the TBARS value in the control group exceeded the acceptable threshold, whereas that in the coating group remained significantly lower throughout storage. This may be attributed to the low temperature combined with the antioxidant properties and low oxygen permeability of the composite emulsion, which effectively inhibited lipid oxidative rancidity. These findings corroborate the metabolomics results and are consistent with the trends reported by Yu et al. (2022) [25].

### 3.5. TVB-N

TVB-N serves as a key freshness indicator for aquatic products [26]. GB 10136-2015 sets 30 mg/100 g as the maximum acceptable TVB-N value for pre-prepared animal aquatic products, except dried and salted items [27], this value was therefore used as the acceptability threshold. As shown in Figure 1D, TVB-N started at 2.10 mg/100 g and then increased significantly during storage (*p* < 0.05). The increase was mainly due to protein degradation by endogenous enzymes and spoilage microorganisms, which generated volatile amines such as cadaverine and putrescine. On Day 7, the control samples reached 31.50 mg/100 g and exceeded the 30 mg/100 g limit, while the coated samples exceeded it only on Day 10. The control group consistently had higher TVB-N than the coated group (*p* < 0.05), consistent with Jiang et al. (2019) [21] for chitosan-based coatings applied to tuna. In summary, application of the CS-LEO/NE-R coating reduced protein breakdown and suppressed the formation of volatile basic nitrogen in seasoned white snakehead fillets, allowing the samples to remain below the TVB-N acceptability threshold for approximately 3 additional days.

### 3.6. TVC

The TVC is a key indicator of microbial growth and spoilage in aquatic products during storage [28]. According to GB 10136-2015, the acceptable TVC limit in animal aquatic products is 5 log CFU/g [27]. As shown in Figure 1E, TVC increased continuously in both groups during storage (*p* < 0.05). The initial counts were less than 4.00 log CFU/g in both groups. By Day 7, the TVC in the control group reached 5.63 log CFU/g and exceeded the national limit, whereas the coating group remained below the limit at 4.98 log CFU/g on Day 10. These results indicate that, compared with controlled freezing-point storage alone, the coating treatment prolonged the microbiological acceptability period by approximately 3 days under the present experimental conditions. The inhibitory effect may be related to the surface barrier formed by the coating and to the antimicrobial activity of lemon essential oil and chitosan, which together reduced exposure to air and limited microbial proliferation [29]. Similar observations were reported by Khorami et al. (2024) [30], who found that chitosan-based bilayer coatings suppressed TVC increases in refrigerated fish fillets.

### 3.7. Sensory Quality Evaluation

**Figure 2:**
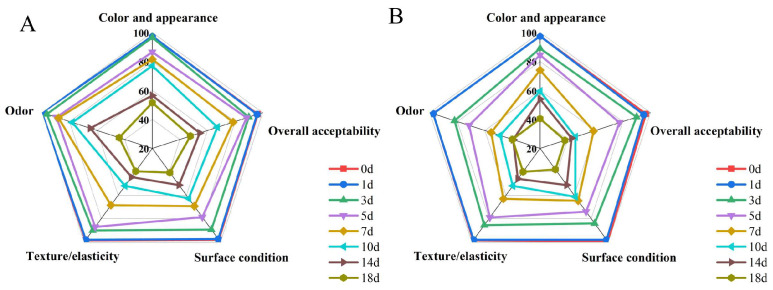
Radar charts showing the changes in quality-related indicators of seasoned northern snakehead fillets during controlled freezing-point storage. (**A**) Coating group; (**B**) Control group.

Sensory evaluation provides a direct reflection of the visual, olfactory, and textural acceptability of fish products during storage [31]. In this experiment, sensory scores declined gradually in both treatments, indicating continuous deterioration of the seasoned fillets. As shown in Figure 2B, the decline was more evident in the control group, which developed a duller surface, more exudate, weaker elasticity, and stronger fishy or unpleasant odors at the later stages. By contrast, the Coating group (Figure 2A) retained higher scores and better overall acceptability, showing that the coating delayed the loss of sensory quality.

These sensory changes were consistent with the variations in TVB-N, TBARS, TVC, and texture properties. The increase in TVB-N reflected the accumulation of volatile nitrogenous compounds caused by protein degradation and microbial activity, which contributed to off-odor development. Meanwhile, the increase in TBARS indicated lipid oxidation, which may be associated with rancid odor and color deterioration. The decrease in hardness, springiness, cohesiveness, and resilience further explained the softening and loss of elasticity perceived by the panelists. Therefore, the better sensory quality of the coated samples was in agreement with their lower TVB-N, TBARS, and TVC values and better retention of texture properties. These findings suggest that CS-LEO/NE-R delayed sensory deterioration by limiting microbial growth, reducing protein and lipid degradation, and preserving muscle structural integrity.

### 3.8. Nontargeted Metabolomics

#### 3.8.1. Metabolite Classification and Principal Component Analysis

**Figure 3:**
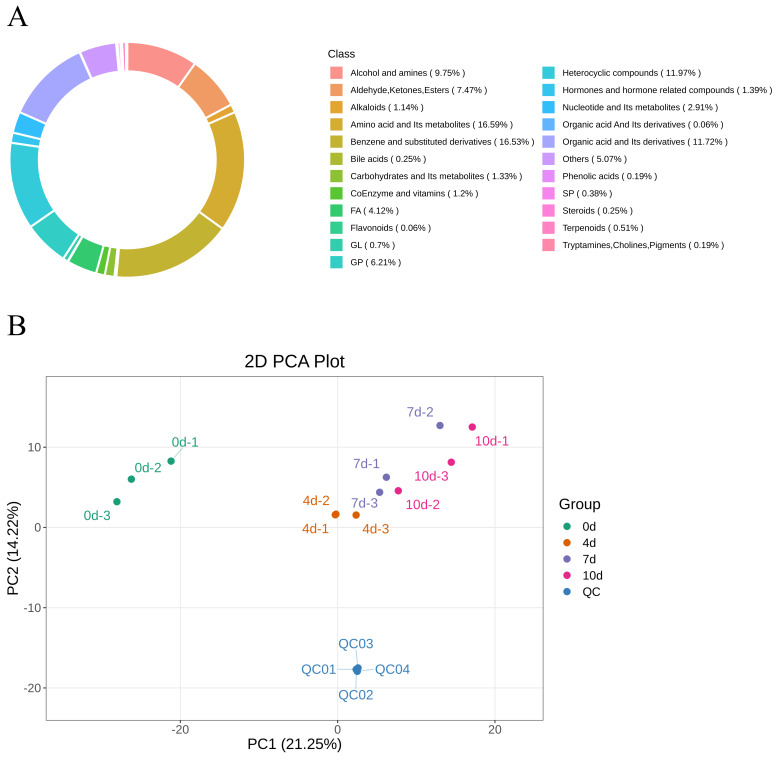
(**A**) Composition and proportion of metabolite categories in seasoned white snakehead fillets. (**B**) PCA analysis of seasoned white snakehead fillet samples.

In this study, 2267 metabolites were identified and annotated. As shown in Figure 3A, the major categories included amino acids and their metabolites (16.59%), benzene and substituted derivatives (16.53%), heterocyclic compounds (11.97%), organic acids and their derivatives (11.72%), alcohols and amines (9.75%), and aldehydes, ketones, and esters (7.47%). Other categories included glycerophospholipids (GP, 6.21%), fatty acyls (FA, 4.12%), nucleotides and their metabolites (2.91%), and others.

Principal component analysis (PCA) characterized the multidimensional metabolomics profiles. A tight cluster of QC samples near the plot center (Figure 3B) indicated that sample extraction and instrumental acquisition introduced only limited error, confirming result reliability [32]. During each storage period, the three biological replicates clustered together, demonstrating good homogeneity among replicates. The first two principal components explained 35.47% of the total variance (PC1 = 21.25%, PC2 = 14.22%). Although this cumulative value was below 70%, this is not unusual for high-dimensional untargeted metabolomics datasets in which variance is distributed across many metabolites. Therefore, PCA was used mainly for exploratory visualization and quality-control assessment rather than as the sole basis for differential metabolite selection. Subsequent OPLS-DA validation and univariate statistics were used together to support the screening of differential metabolites.

#### 3.8.2. Orthogonal Partial Least Squares Discriminant Analysis (OPLS-DA)

**Figure 4:**
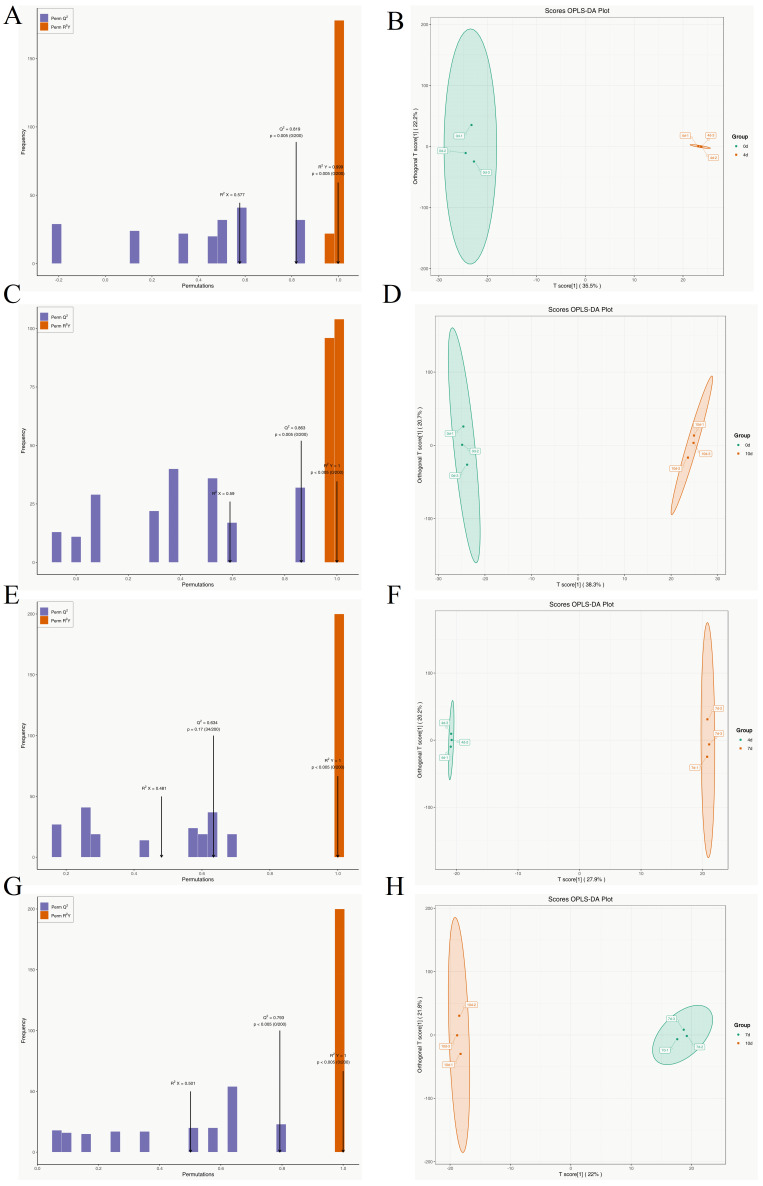
Plots of OPLS-DA scores from two-by-two comparisons for differentially enriched metabolites (**A**,**C**,**E**,**G**) and model validation (**B**,**D**,**F**,**H**). (**A**,**B**) 0 day vs. 4 day; (**C**,**D**) 0 day vs. 10 day; (**E**,**F**) 4 day vs. 7 day; (**G**,**H**) 7 day vs. 10 day.

In OPLS-DA, R^2^X and R^2^Y represent the variance explained in the X and Y matrices, and Q^2^ reflects the model’s predictive ability [33]. Generally, Q^2^ > 0.5 indicates acceptable predictive performance, and higher R^2^Y and Q^2^ values indicate better model fitness and prediction [34]. As shown in Figure 4A–H, the samples from different storage time points were clearly separated in the OPLS-DA score plots, indicating obvious metabolomic differences among seasoned white snakehead fillets stored for different durations, which was consistent with the PCA results. The OPLS-DA models were further evaluated using 200 permutation tests. The Q^2^ values of the four models ranged from 0.634 to 0.863, with R^2^Y values close to 1, and the permutation test results showed statistical significance (*p* < 0.05). These results suggested that the OPLS-DA models had acceptable predictive ability and no obvious overfitting tendency based on the available validation parameters. Nevertheless, because OPLS-DA is a supervised method, the model results were interpreted together with PCA and univariate statistical analysis, and differential metabolites were screened using both VIP > 1 and *p* < 0.05.

#### 3.8.3. Analysis of Significantly Different Enriched Metabolites

**Figure 5:**
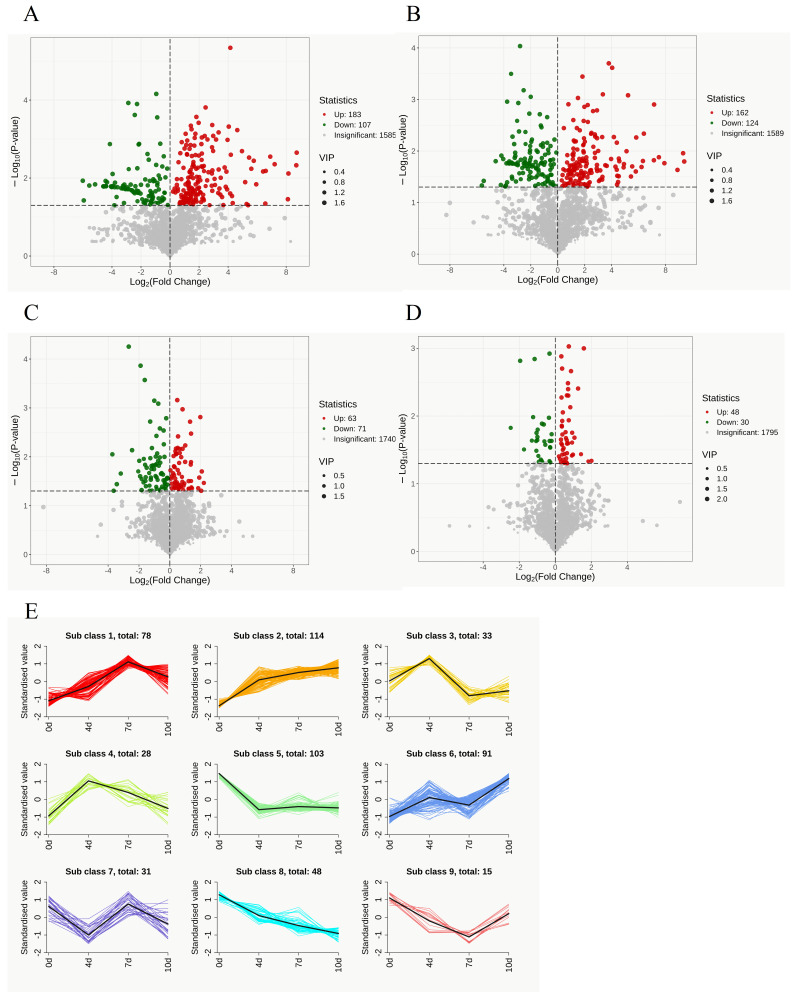
(**A**–**D**) Differential substance metabolism volcano plots of differential metabolites for the four comparison groups: (**A**) 0 day vs. 4 day, (**B**) 0 day vs. 10 day, (**C**) 4 day vs. 7 day, (**D**) 7 day vs. 10 day. (**E**) K-means clustering analysis plot of differential metabolites.

To screen for differentially abundant metabolites, variable importance in projection (VIP) values from OPLS-DA were combined with univariate statistical analysis [35]. In this study, metabolites were considered differentially abundant when VIP > 1.0 and *p* < 0.05. As shown in Figure 5A–D, 290 metabolites differed between 0 day and 4 day, including 183 increased and 107 decreased features; 286 metabolites differed between 0 day and 10 day, including 162 increased and 124 decreased features; 134 metabolites differed between 4 day and 7 day, including 63 increased and 71 decreased features; and 78 metabolites differed between 7 day and 10 day, including 48 increased and 30 decreased features. The relatively smaller number of differential features between 7 day and 10 day suggests that metabolite changes became less extensive in the later stage under the composite preservation condition [36]. This trend may be related to slower biochemical deterioration, consistent with the delayed increases in TBARS, TVB-N, and TVC observed in the coated samples, but it should not be interpreted as complete metabolic stabilization.

To further elucidate the dynamic changes in the differentially abundant metabolites of the samples during storage, a K-means clustering algorithm was used for temporal pattern analysis of the differentially abundant metabolites. As shown in Figure 5E, on the basis of the accumulation patterns of metabolites, the metabolites were classified into nine characteristic clusters. Among these, 61 metabolites (Subclasses 3 and 4) peaked at 4 day, whereas 109 metabolites (Subclasses 1 and 7) peaked at 7 day, indicating relatively high abundance during the midstorage period. A total of 205 metabolites (Subclasses 2 and 6) tended to increase overall throughout the entire storage period, whereas 103 metabolites (Subclass 5) did not significantly differ among 4 day, 7 day, and 10 day. Additionally, 48 metabolites (Subclass 8) tended to decrease during storage, whereas 15 metabolites (Subclass 9) were highly abundant at 0 d but subsequently decreased rapidly, reaching very low levels by 7 day, which may reflect the consumption or transformation of freshness-related substrates. These temporal patterns indicate staged changes in metabolite abundance during storage. Metabolites with increasing trends may include products associated with lipid oxidation, proteolysis, microbial metabolism, or coating-derived compounds, whereas decreasing metabolites may represent substrates that were consumed or transformed [8].

On the basis of the stage-specific characteristics described above, the top 20 differentially abundant metabolites ranked by their VIP values in each comparison group were analyzed. The results showed that these core metabolites exhibited distinct stage specificity and mutually corroborated the trends observed in the K-means clustering analysis.

Comparing 0 day and 4 day, the upregulated metabolites with the highest VIP values primarily included Gly-Asp-Ile-Val-Ile and prosapogenin A. The upregulation of peptide fragments and saponin-like or plant-derived annotations may be associated with early changes in protein fragments and the presence or interaction of coating-related compounds [37]. The concomitant downregulation of several amino acids and dihydrogeranylgeranyl diphosphate may reflect consumption or transformation of freshness-related and terpenoid-associated substrates during early storage [8].

For the 0 day and 10 day comparison, the metabolite profile further changed. Carbenoxolone, peptides, and the lipid-derived molecule tumonoic acid I were significantly upregulated, while prosapogenin A remained upregulated. These changes suggest the accumulation or transformation of peptide, lipid, and plant-derived annotated compounds during prolonged storage. Downregulated metabolites such as thymopentin, dichloroacetic acid, azocyclotin, progesterone 3-biotin, and citrulline may be associated with changes in nitrogen-containing compounds, protein degradation, and microbial metabolism [15,17].

When comparing 4 day and 7 day, the core metabolite spectrum shifted markedly. Metabolites such as choline, 3-(pyrimidin-2-yl) propanoic acid and 2-hydroxy-3-(phosphonooxy)propyl stearate were significantly upregulated. The enrichment of choline and phospholipid-related compounds may be related to membrane lipid rearrangement or degradation during storage [38]. The downregulation of metabolites, including the long-chain fatty acid derivative 17-(4-hydroxyphenyl)heptadecanoic acid, 9-fluorenone, the energy carrier inosine diphosphate, and quinaldic acid, may indicate changes in fatty acid-derived compounds, energy-related nucleotides, and microbial metabolites at this stage [36].

Between 7 day and 10 day, the upregulated metabolites were mainly represented by the salicylic acid derivative amino salicylic acid, the flavor compound methylpyrazine, the short peptides Pro-Ala and Tyr-Ser-Asn, and the energy metabolism-related guanidino compound glycocyamine. The accumulation of methylpyrazine and small peptides may be associated with the development of flavor-active nitrogen-containing compounds and protein-derived fragments during late storage. Glycocyamine and related guanidino compounds may indicate changes in nitrogen metabolism, whereas the downregulation of xylitol and all-trans-decaprenyl diphosphate suggests further transformation of carbohydrate-related and long-chain isoprenoid-associated compounds [14].

#### 3.8.4. Correlation Network of Core Metabolites

**Figure 6:**
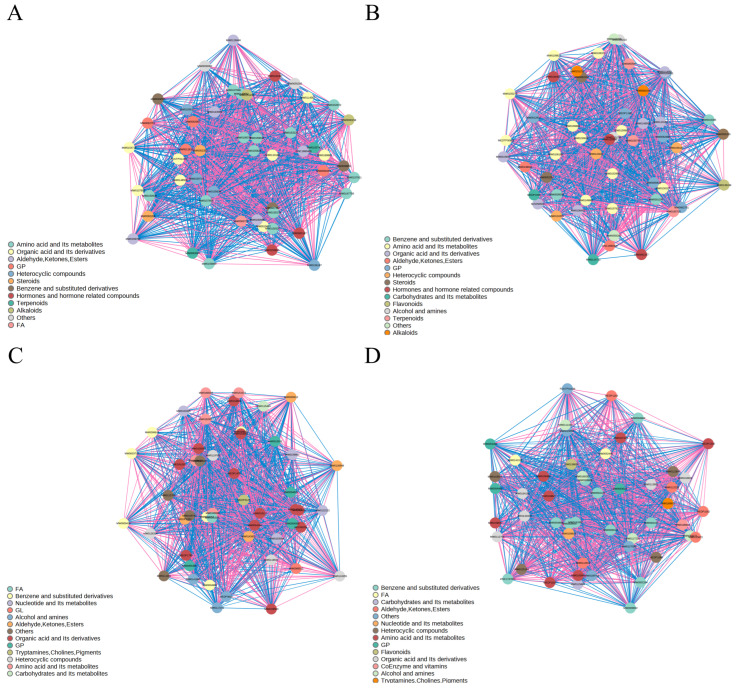
(**A**–**D**) Differentially abundant metabolite chord plots for the four comparison groups: (**A**) 0 day vs. 4 day, (**B**) 0 day vs. 10 day, (**C**) 4 day vs. 7 day, (**D**) 7 day vs. 10 day.

Correlation network analysis was performed using the top 50 metabolites based on VIP values. As shown in Figure 6, each node corresponds to one metabolite, and the node size reflects its connectivity (larger nodes indicate more connections) [39].

When comparing 0 day and 4 day, the correlation network of differentially abundant metabolites showed a clear co-variation pattern. The significantly upregulated Gly-Asp-Ile-Val-Ile showed a strong positive correlation with the likewise upregulated prosapogenin A, which may indicate co-accumulation of peptide fragments and saponin-like or plant-derived annotated compounds in the early stage. In contrast, Gly-Asp-Ile-Val-Ile was negatively correlated with psychotridine, and prosapogenin A was negatively correlated with the terpenoid precursor dihydrogeranylgeranyl diphosphate. These opposite trends suggest that alkaloid-like and terpenoid-associated annotations changed in different directions during early storage. These correlations should be considered evidence of co-variation among detected features rather than proof of direct biochemical regulation [40].

Across the 0–10 days period, the correlation network of differentially abundant metabolites underwent further changes. The consistently upregulated tripeptide L-seryl-L-lysyl-L-lysine was positively correlated with the lipid-derived metabolite 9alpha,11alpha,15R-trihydroxy-17-phenyl-18,19,20-trinor-prost-5Z-en-1-oic acid isopropyl ester, but negatively correlated with carbenoxolone and prosapogenin A. These correlations may reflect differential accumulation of peptide-, lipid-, and plant-derived annotated compounds during prolonged storage. Because the samples were postmortem fish fillets, the network should not be interpreted as anti-inflammatory or stress-defense crosstalk; instead, it indicates coordinated or opposing changes among metabolite features that may be linked to lipid oxidation and proteolysis. These metabolite trends were consistent with the increases in TBARS and TVB-N observed during the same period, further supporting their association with lipid oxidation and protein degradation [41].

In the 4 day and 7 day groups, the composition of the core nodes in the correlation network of differentially abundant metabolites shifted. 17-(4-Hydroxyphenyl)heptadecanoic acid was negatively correlated with the sterol metabolic intermediate 7alpha,12alpha-dihydroxy-5alpha-cholestan-3-one but positively correlated with 1,6-di-O-phosphono-D-fructose. Quinaldic acid was negatively correlated with the energy carrier inosine diphosphate (IDP) but positively correlated with [(2R,4S,5R)-4-hydroxy-5-(phosphonooxymethyl)oxolan-2-yl] dihydrogen phosphate. These correlations suggest associations among membrane lipid changes, sugar-phosphate intermediates, energy-related metabolites, and microbial or tryptophan-derived alkaloid-like annotations. Salicin 6-phosphate was positively correlated with both IDP and quinaldic acid, further indicating that glycoside metabolism, energy carriers, and nitrogen-containing metabolites may have changed together during mid-storage [42].

For the 7 day and 10 day comparison, the network exhibited a predominantly positive co-variation pattern. The upregulated key metabolites identified in the VIP analysis, such as methylpyrazine, the short peptides Pro-Ala and Tyr-Ser-Asn, and aminosalicylic acid, were significantly positively correlated with one another. This pattern indicates co-accumulation of flavor-active and peptide-related metabolites. Together with the smaller number of differential metabolites at this stage, the network suggests that late-stage changes were concentrated in nitrogen-containing and flavor-related compounds [43].

#### 3.8.5. KEGG Enrichment Analysis

**Figure 7:**
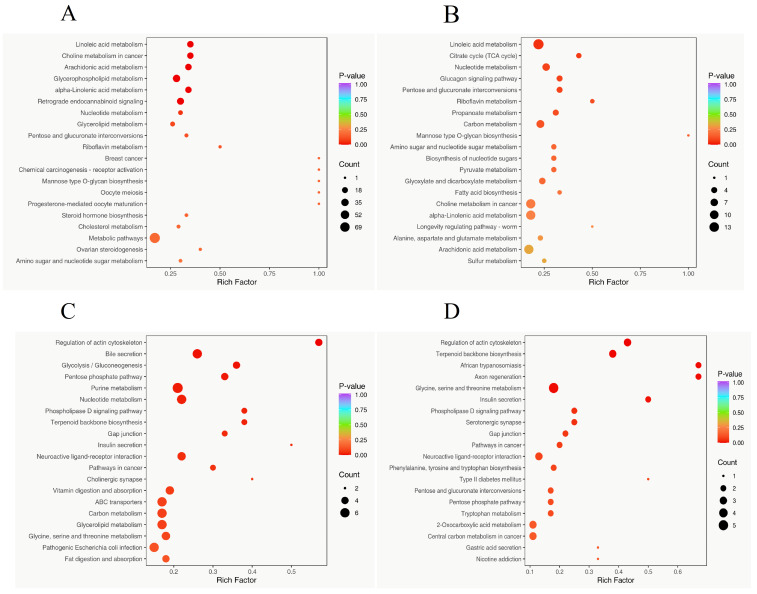
(**A**–**D**) Differentially abundant metabolite pathway enrichment maps for the four comparison groups: (**A**) 0 day vs. 4 day, (**B**) 0 day vs. 10 day, (**C**) 4 day vs. 7 day, (**D**) 7 day vs. 10 day.

KEGG pathway enrichment analysis was performed based on the differentially abundant metabolites, and bubble plots were generated for enriched pathways [9]. The closer the *p* value is to 0, the more significant the enrichment [44]. As shown in Figure 7, throughout the entire storage process, the 10 most significantly enriched KEGG categories for differentially abundant metabolites were linoleic acid metabolism, choline metabolism in cancer, arachidonic acid metabolism, glycerophospholipid metabolism, alpha-linolenic acid metabolism, retrograde endocannabinoid signaling, regulation of the actin cytoskeleton, terpenoid backbone biosynthesis, axon regeneration, and African trypanosomiasis. It should be emphasized that disease or hormone-related KEGG categories, such as choline metabolism in cancer, axon regeneration, African trypanosomiasis, glucagon signaling, or insulin secretion, were treated only as database annotation outputs. In the context of postmortem fish fillets, these terms were used to organize structurally related metabolites rather than to infer corresponding physiological processes.

KEGG pathway enrichment analysis revealed 459 differentially abundant metabolites between 0 day and 4 day, which were annotated to 99 KEGG pathways. Among these pathways, linoleic acid metabolism, arachidonic acid metabolism, glycerophospholipid metabolism, alpha-linolenic acid metabolism, retrograde endocannabinoid signaling, and metabolic pathways were significantly enriched. Glycerophospholipid metabolism included the largest number of differentially abundant metabolites, with a total of 35 metabolites. The enrichment of glycerophospholipid metabolism and unsaturated fatty acid-related pathways was consistent with the increase in TBARS during storage, indicating that lipid oxidation and membrane lipid transformation were important contributors to quality deterioration [45]. Compared with controlled freezing-point storage alone, the lower TBARS value in the coated fillets under controlled freezing-point storage (0.87 mg/kg vs. 1.53 mg/kg at the corresponding stage) suggests that the CS-LEO/NE-R coating delayed lipid oxidation under ice-temperature conditions. This effect may be related to the oxygen-barrier property of chitosan and the antioxidant capacities of lemon essential oil and rutin, such as free radical scavenging and metal chelation, as described in previous studies [25]. Thus, the lipid pathway results provide a biochemical explanation for the slower oxidative deterioration observed in the coated fillets stored under controlled freezing-point conditions.

A comparison between 0 day and 10 day yielded 336 differentially abundant metabolites that were assigned to 82 KEGG pathways. In addition to the consistently enriched linoleic acid metabolism, pathways related to energy and primary metabolism, such as the citrate cycle (TCA cycle), carbon metabolism, and nucleotide metabolism, were enriched. These pathways may reflect continued substrate breakdown and turnover during storage rather than active metabolic adaptation. Changes in the TCA cycle, carbon metabolism, and nucleotide metabolism are consistent with postmortem biochemical reactions, protein degradation, and microbial activity, which also contributed to increases in TVB-N and pH.

In the comparison between 4 day and 7 day, 271 differentially abundant metabolites were identified and annotated to 105 KEGG pathways, with a notable shift in enriched categories. Pathways related to cellular structure, substance transport, and signal transduction, including regulation of the actin cytoskeleton, bile secretion, glycolysis/gluconeogenesis, the pentose phosphate pathway, purine metabolism, nucleotide metabolism, terpenoid backbone biosynthesis, and the phospholipase D signaling pathway, were significantly enriched [46]. In this stage, enrichment of the regulation of the actin cytoskeleton and phospholipase D signaling pathway was consistent with the progressive decline in texture parameters and may be associated with changes in structural proteins, membrane lipids, and cell–matrix integrity during storage. Because the coating contained lemon essential oil, terpenoid-related annotations may partly originate from exogenous coating-derived compounds or their interaction with fish muscle components, rather than from enhanced endogenous terpenoid synthesis [47].

This trend was extended in the comparison between 7 day and 10 day. Pathways related to regulation of the actin cytoskeleton, terpenoid backbone biosynthesis, and the phospholipase D signaling pathway remained enriched, while amino acid-related categories, such as glycine, serine and threonine metabolism, also appeared. Glycine, serine and threonine metabolism was the top pathway in terms of enriched differential metabolites, with five such metabolites. The enrichment of amino acid-related pathways was consistent with the accumulation of short peptides and nitrogen-containing compounds in the VIP and network analyses. These changes may explain the continued increase in TVB-N and the formation of flavor-active molecules such as methylpyrazine.

Comparisons across different storage stages showed that several KEGG pathway annotations were repeatedly enriched during storage, including linoleic acid metabolism, terpenoid backbone biosynthesis, regulation of the actin cytoskeleton, and the phospholipase D signaling pathway. These recurrently enriched pathways suggest that lipid oxidation, terpenoid-related compounds, membrane-associated alterations, and structural deterioration were important biochemical features of the preservation process [48]. However, these metabolomic alterations should not be attributed solely to the coating treatment. Because the coated samples were stored under controlled freezing-point conditions, the observed metabolic changes more likely reflected the combined effects of coating-derived components, postmortem biochemical reactions, microbial activity, lipid oxidation, and their interactions during storage. In particular, terpenoid-related annotations may partly originate from lemon essential oil-derived compounds or their transformation and interaction products, whereas lipid- and membrane-associated pathways may be linked to storage-induced oxidation, phospho-lipid degradation, membrane integrity loss, and tissue deterioration. The enrichment of regulation of the actin cytoskeleton and phospholipase D signaling pathway may provide a metabolite-level explanation for the slower decline in hardness and springiness in the coated group, as membrane integrity and structural stability are closely associated with water retention and texture maintenance in muscle foods [9]. Nevertheless, these KEGG categories should be interpreted as pathway-level associations of altered metabolites rather than direct evidence of active cellular signaling in postmortem fish tissue. Further validation using targeted metabolomics, enzyme activity assays, coating migration analysis, and microbial community analysis is required to distinguish coating-derived metabolites from storage-induced metabolic changes.

## 4. Conclusions

In this study, applying the CS-LEO/NE-R coating during controlled freezing-point storage effectively slowed the decline in the overall quality of seasoned white snakehead fillets. This protective effect was reflected by more moderate increases in TBARS, TVB-N, and TVC, together with improved preservation of textural characteristics. Under the present experimental conditions, the coating prolonged the microbiological and physicochemical acceptability period by approximately 3 days compared with controlled freezing-point storage alone. Untargeted metabolomics revealed 2267 metabolites, and the differentially abundant ones mainly comprised amino acids, heterocyclic compounds, aldehydes, ketones, esters, and lipid-related metabolites. KEGG enrichment suggested that changes in linoleic acid metabolism, terpenoid-related annotations, the actin cytoskeleton, and the phospholipase D signaling pathway were associated with delayed quality deterioration. Further studies integrating proteomics, microbiomics, and other omics approaches will be useful for clarifying the preservation mechanism of this composite coating system.

## Figures and Tables

**Table 1:** Texture properties of seasoned white snakehead fillets during controlled freezing-point storage under different treatments.

		Storage Time (day)							
		0	1	3	5	7	10	14	18
Hardness/g	Control	6325.6 ± 1213.21 ^a^	5976.02 ± 619.42 ^ab^	5672.57 ± 687.16 ^ab^	5074.80 ± 1072.84 ^ab^	4742.85 ± 205.57 ^ab^	4062.36 ± 2278.94 ^ab^	3731.06 ± 700.01 ^b^	3672.64 ± 443.72 ^b^
	Coating	6325.6 ± 1213.21 ^a^	5398.59 ± 654.26 ^ab^	5346.57 ± 922.14 ^ab^	5435.23 ± 242.54 ^ab^	4328.65 ± 1778.67 ^ab^	4822.33 ± 2607.87 ^ab^	4309.57 ± 768.06 ^ab^	4028.79 ± 386.78 ^ab^
Springiness	Control	0.62 ± 0.01 ^ab^	0.65 ± 0.04 ^a^	0.7 ± 0.17 ^a^	0.64 ± 0.11 ^a^	0.62 ± 0.14 ^ab^	0.57 ± 0.08 ^ab^	0.54 ± 0.06 ^ab^	0.44 ± 0.21 ^b^
	Coating	0.62 ± 0.02 ^ab^	0.71 ± 0.09 ^a^	0.71 ± 0.07 ^a^	0.69 ± 0.03 ^a^	0.61 ± 0.09 ^ab^	0.59 ± 0.06 ^ab^	0.57 ± 0.06 ^ab^	0.58 ± 0.02 ^ab^
Cohesiveness	Control	0.65 ± 0.01 ^ab^	0.76 ± 0.04 ^a^	0.62 ± 0.04 ^ab^	0.71 ± 0.13 ^ab^	0.64 ± 0.02 ^a^	0.66 ± 0.09 ^ab^	0.63 ± 0.14 ^ab^	0.46 ± 0.08 ^c^
	Coating	0.65 ± 0.02 ^a^	0.69 ± 0.03 ^a^	0.61 ± 0.03 ^a^	0.69 ± 0.15 ^ab^	0.64 ± 0.05 ^a^	0.64 ± 0.04 ^ab^	0.62 ± 0.02 ^ab^	0.58 ± 0.04 ^a^
Chewiness/mJ	Control	2035.08 ± 87.58 ^a^	2422.93 ± 191.50 ^a^	2268.94 ± 186.75 ^a^	1933.43 ± 174.27 ^b^	1877.61 ± 125.68 ^b^	1667.29 ± 508.69 ^b^	1511.87 ± 484.08 ^b^	1370.62 ± 611.11 ^b^
	Coating	2035.08 ± 87.58 ^a^	2556.96 ± 369.39 ^a^	2513.38 ± 499.12 ^a^	2116.61 ± 569.98 ^a^	2092.14 ± 919.51 ^a^	2077.18 ± 955.13 ^a^	2020.27 ± 1342.34 ^a^	1863.66 ± 579.32 ^a^
Resilience	Control	0.54 ± 0.05 ^b^	0.7 ± 0.09 ^a^	0.55 ± 0.08 ^b^	0.53 ± 0.08 ^bc^	0.51 ± 0.01 ^bc^	0.48 ± 0.02 ^bc^	0.41 ± 0.02 ^c^	0.36 ± 0.06 ^d^
	Coating	0.54 ± 0.05 ^a^	0.57 ± 0.14 ^a^	0.56 ± 0.05 ^a^	0.52 ± 0.03 ^a^	0.5 ± 0.05 ^a^	0.52 ± 0.03 ^a^	0.55 ± 0.03 ^a^	0.51 ± 0.02 ^a^

Note: Different lowercase letters indicate significant differences among storage times within the same treatment (*p* < 0.05).

## Data Availability

The original contributions presented in this study are included in the article. Further inquiries can be directed to the corresponding authors.
